# Prognostic nomogram predicts overall survival in pulmonary large cell neuroendocrine carcinoma

**DOI:** 10.1371/journal.pone.0223275

**Published:** 2019-09-27

**Authors:** Yanqi He, Han Liu, Shuai Wang, Yu Chen

**Affiliations:** 1 Department of Respiratory and Critical Care Medicine, West China Hospital, Sichuan University, Chengdu, China; 2 Department of Respiratory Medicine, the First Hospital of Jilin University, Changchun, China; 3 Department of Vascular Surgery, the First Hospital of Jilin University, Changchun, China; 4 Department of Cardiology, Hospital of The University of Electronic Science and Technology of China and Sichuan Provincial People's Hospital, Chengdu, China; University of Nebraska Medical Center, UNITED STATES

## Abstract

**Background:**

Large cell neuroendocrine carcinoma (LCNEC) is a rare and typically aggressive malignancy with poor prognosis. This study developed a nomogram model to predict the overall survival (OS) of patients with LCNEC.

**Methods:**

LCNEC patients were identified from the Surveillance, Epidemiology, and End Results database between 2004–2014. Univariate and multivariate Cox regression models were used to determine demographic and clinicopathological features associated with OS. A nomogram model was generated to predict OS and its performance was assessed by Harrell’s concordance index (C-index), calibration plots, and subgroup analysis by risk scores.

**Results:**

Of 3048 eligible patients with LCNEC, 2138 were randomly grouped into the training set and 910 into the validation set. Age at diagnosis, gender, tumor stage, N stage, tumor size, and surgery of primary site were independent prognostic factors of OS. C-index values of the nomogram were 0.75 (95% CI, 0.74–0.76) and 0.76 (95% CI, 0.74–0.77) in the training and validation sets, respectively. In both cohorts, the calibration plots showed good concordance between the predicted and observed OS at 3 and 5 years. Kaplan-Meier curves revealed significant differences in OS in patients stratified by nomogram-based risk score, and patients with a higher-than-median risk score had poorer OS.

**Conclusion:**

This is the first nomogram developed and validated in a large population-based cohort for predicting OS in patients with LCNEC, and it shows favorable discrimination and calibration abilities. Use of this proposed nomogram has the potential to improve prediction of survival risk, and lead to individualized clinical decisions for LCNEC.

## Introduction

Large cell neuroendocrine carcinoma (LCNEC) of the lung is a rare malignant tumor accounting for only 2 to 3% of all primary lung cancers and carries an aggressive clinical behavior and poor prognosis [1]. Although previously regarded as a subgroup of large cell carcinoma [2], LCNEC was pathologically reclassified in 2015 as a high-grade neuroendocrine tumor, comprising the subgroups small cell lung cancer, typical carcinoid, and atypical carcinoid [3]. LCNEC often exhibits large cell morphology and features of neuroendocrine differentiation, including high mitotic rate (>10 mitoses per 10 high-power fields), low nuclear-to-cytoplasm ratio, and frequent areas of necrosis. Old men (median age 65 years) exposed to heavy smoking are the known risk factors for LCNEC occurrence [4].

Estimating the prognosis of LCNEC remains challenging, due to the complex and heterogeneous biological behaviors of the disease. For example, patients with LCNEC within the same stage I disease have demonstrated different survival patterns; the worst prognosis is shown by Caucasians, older than 70 years, with comorbid conditions, and undergoing sublobar resections for large tumors (>20 mm) [5]. Although small cell lung cancer is considered the same clinical entity and treated with the conventional chemotherapeutic regimen, these patients are heterogeneous in terms of survival, and no accurate indicator of prognosis has been established [6]. Identification of patients at clinical high risk with homogenous prognosis is crucial for effective clinical practice, decision-making, and clinical trial.

The most widely used staging system for LCNEC is the tumor-node-metastasis (TNM) classification released by the International Association for Study of Lung Cancer International (IASLC) Staging Project [7]. Yet, TNM staging is not perfect for predicting prognosis of LCNEC, because it does not take into consideration tumor size, metastasis sites/patterns, and pathological, genetic, and therapeutic factors associated with different prognosis. Cattoni et al. reported that the pathologic tumor stage, but not cancer grade, was independently associated with survival of LCNEC [8]. Qiao et al. demonstrated that patients with LCNEC with metastasis, especially to the liver metastasis or to multiple organs, had poor prognosis regarding overall survival (OS) and lung cancer-specific survival [9]. Kujtan L et al conducted a retrospective evaluation of LCNEC patients with surgically resected stage I disease and found that improved survival was achieved by chemotherapy in both stage IA and IB patients [5]. There is a need for studies to comprehensively analyze prognostic factors and develop a model to predict, precisely and accurately, survival for the individual patient with LCNEC.

Nomograms are widely used in the field of oncology as prognostic tools to predict the probability of disease outcomes with a simple and straightforward figure that integrate the relevant variables in complex mathematical models [10]. Nomograms can improve discriminatory accuracy and prediction of outcomes [11], and have been used successfully to quantify risk for various malignancies [12, 13]. However, a nomogram has not been developed for patients with LCNEC. Given the limitations of TNM staging for LCNEC prognosis, developing a superior and accurate prognostic indicator has clinical importance for guiding personalized cancer therapy.

The present study developed an elaborate nomogram to estimate individualized prognosis for LCNEC in terms of 3-year and 5-year OS using the Surveillance Epidemiology, and End Results (SEER) data, a large population-based cancer cohort with a coverage of approximately 28% of the United States population [14].

## Patients and methods

No written informed consent was obtained for this study because the data were de-identified and publicly available.

### Data sources and patient selection

We retrieved data from 18 population-based cancer registries in the SEER program using the SEER*Stat software (version 8.3.5) [15]. The diagnosis of LCNEC was defined by the International Classification of Diseases for Oncology, Third Edition (ICD-O-3) histology code (8013) and site recode (lung and bronchus, trachea, mediastinum and other respiratory organs). The inclusion criteria were as follows: diagnosed between 2000 and 2014, and first and only primary cancer diagnosis. Autopsy only and death certification only cases were excluded. These selection criteria resulted in 3038 eligible patients with LCNEC in the primary cohort. These patients in the primary cohort were subsequently randomly assigned, using a simple random splitting method, in a ratio of 7 to 3 to the training or validation sets, using R version 3.5.1 and the “caret” package.

### Study variables

The following data were obtained from the SEER database: demographics (gender, race, age at diagnosis, and year at diagnosis); tumor characteristics (primary site, laterality, histologic grade, tumor size, T, N, and M stages, separate tumor nodules-ipsilateral lung); and treatment information (surgery to other reginal/distant sites, and surgery to primary site). Race in SEER is coded as white, black, American Indian/Alaskan, Asian/Pacific Islander, and unknown. Due to the small sample size of the last three categories, the American Indian/Alaskan, Asian/Pacific Islander, and unknown races were grouped together as “other” in analysis. The primary outcome was OS, defined as the time between diagnosis and death of any cause. Patients still alive on December 31, 2015 were censored based on vital status recode in the SEER database. Surgery of primary cancer and other regional/distant sites were divided into two categories (yes and no).

### Statistical analyses

Frequency distributions of demographic, clinical, and pathologic characteristics of the eligible patients were compared between the training and validation sets using the chi-square test. In the training cohort, univariate and multivariate Cox proportional hazards regression models were applied to calculate hazard ratios (HRs) and corresponding 95% confidence intervals (CIs) for demographic and clinical factors as predictors of OS. Variables subject to univariate analysis included age at diagnosis (<50, 50–59, 60–69, 70–79, and ≥80 years), race (black, white, and others), gender (female and male), primary site (main bronchus, upper lobe, middle lobe, lower lobe, overlapping lesion of lung, and lung NOS), laterality (left vs right vs others), histologic grade (I, II, III, IV, and unknown), tumor stage (I, II, III, and IV), T stage (T1, T2, T3, T4, and Tx), N stage (N0, N1, N2, N3, and Nx), M stage (M0, M1a, M1b, and M1 NOS), tumor size (<20, 20–29 30–39 40–49, and ≥50 mm), separate tumor nodules—ipsilateral lung (no separate nodules, in different lobe, in the same lobe, separate tumor nodules NOS, and unknown), surgery to other regional/distant Sites (yes and no), surgery of primary site (yes and no). Missing data were grouped as a separate category in the regression analysis. Significant variables (*P* < 0.05) in the univariate Cox regression analyses were included in the multivariate Cox regression analysis. The variables retained as significant in the multivariate model were incorporated into the nomogram.

Three-year and 5-year OS rates were the primary endpoints of this study. To test prognostic accuracy, the nomogram model was assessed by adequate discrimination and calibration with bootstrapping sampling in the validation set [16]. Discrimination was quantified by Harrell’s concordance index (C-index), in which a value close to absolute 1 indicated a strong predictive ability of the nomogram model. Calibration plots based on Hosmer-Lemeshow goodness-of-fit test were developed to evaluate the predictive accuracy and show the concordance between the predicted and the observed ongoing survival probabilities of patients. In addition, patients were assigned to different risk categories based on their predicted risk scores. Kaplan-Meier survival curves by risk group and other significant predictive factors were calculated and compared using the log-rank test to determine the discriminative significance of the nomogram developed in the study. A 2-sided *P* < 0.05 was considered significant. All statistical analyses were performed using R version 3.5.1 software with the “rms”, “foreign” and “survival” packages.

## Results

### Baseline characteristics of patients

During the years 2004 through 2014, 3048 patients with LCNEC were identified from the SEER database. Of these patients, 2138 and 910 were assigned as the training and validation cohorts, respectively. Table 1 shows the descriptive statistics for the patients in the training and validation cohorts. In general, patients in the training and validation cohorts were comparable in terms of demographic and clinicopathological features. More than half of the patients were men (training vs validation sets, 53.37% vs 56.26%). More than 70% of the cases were diagnosed at age older than 60 years. The majority of the patients were whites (training vs validation, 84.52% vs 84.72%). The most common primary sites were the upper lobe (training vs validation, 58.75% vs 59.34%), and lower lobe (training vs validation, 27.22% vs 25.49%). Almost half of the patients’ disease were of unknown grade (training vs validation, 49.20% vs 46.37%), followed by poorly differentiated grade (training vs validation, 36.58% vs 39.78%). The most frequent tumor size was ≥50 mm, in 32.60% and 33.19% of those in the training and validation cohorts, respectively. More than 40% of the patients in both cohorts had no separate tumor nodules in the ipsilateral lung. In both cohorts, more than 50% received primary site surgery, and fewer than 10% received surgery to other regional and distant sites.

**Table 1:** Demographic, clinical and pathologic characteristics of patients with large cell neuroendocrine carcinoma (LCNEC) of the lung from the SEER 18 database, 2004–2015 (*n* = 3048).

Characteristics	Training Set, n (%)	Validation Set, n (%)
Total	2138 (100)	910 (100)
**Age at diagnosis**, year		
< 50	117 (5.47)	61 (6.70)
50–59	417 (19.51)	171 (18.79)
60–69	733 (34.28)	298 (32.75)
70–79	656 (30.68)	285 (31.32)
>80	215 (10.06)	95 (10.44)
**Gender**		
Female	997 (46.63)	398 (43.74)
Male	1141 (53.37)	512 (56.26)
**Race**		
Black	248 (11.60)	105 (11.54)
White	1807(84.52)	771 (84.72)
Other	83 (3.88)	34 (3.74)
**Primary site**		
Main bronchus	80 (3.74)	30 (3.30)
Upper lobe	1256 (58.75)	540 (59.34)
Middle lobe	90 (4.21)	46 (5.05)
Lower lobe	582 (27.22)	232 (25.49)
Overlapping lesion of lung	23 (1.08)	9 (0.99)
Lung, NOS	107 (5.00)	53 (5.83)
**Histologic grade**		
I (Well differentiated)	12 (0.56)	2 (0.22)
II (Moderately differentiated)	37 (1.73)	10 (1.10)
III (Poorly differentiated)	782 (36.58)	362 (39.78)
IV (Undifferentiated; anaplastic)	255 (11.93)	114 (12.53)
Unknown	1052 (49.20)	422 (46.37)
**Laterality**		
Left	881 (41.21)	365 (40.11)
Right	1236 (57.81)	533 (58.57)
Other	21 (0.98)	12(1.32)
**Tumor Stage**		
I	612 (28.63)	251 (27.58)
II	201 (9.40)	94(10.33)
III	412 (19.27)	173 (19.01)
IV	913 (42.70)	392 (43.08)
**T Stage**		
T1	569 (26.62)	264 (29.01)
T2	724 (33.86)	299 (32.86)
T3	301 (14.08)	138 (15.16)
T4	492 (23.01)	191 (20.99)
Tx	52 (2.43)	18 (1.98)
**N Stage**		
N0	981 (45.88)	402 (44.18)
N1	210 (9.82)	99 (10.88)
N2	671 (31.38)	282 (30.99)
N3	237 (11.10)	111 (12.20)
Nx	39 (1.82)	16 (1.75)
**M Stage**		
M0	1225 (57.30)	518 (56.92)
M1a	98 (4.58)	46 (5.05)
M1b	766 (35.83)	322 (35.38)
M1, NOS	49 (2.29)	24 (2.65)
**Tumor Size**		
<20 mm	409 (19.13)	178 (19.56)
20–29 mm	419 (19.60)	182 (20.00)
30–39 mm	353 (16.51)	152 (16.70)
40-49mm	260 (12.25)	96 (10.55)
≥50 mm	697 (32.60)	302 (33.19)
**Separate Tumor Nodules—Ipsilateral Lung**		
No separate tumor nodules	1000 (46.77)	435 (47.80)
Separate tumor nodules in different lobe of ipsilateral lung	82 (3.84)	28 (3.08)
Separate tumor nodules in same lobe of ipsilateral lung	121 (5.66)	57 (6.26)
Separate tumor nodules, NOS	75 (3.51)	30 (3.30)
Unknown	860 (40.22)	360 (39.56)
**Surgery to Other Regional/Distant Sites**		
No	1972 (92.24)	839 (92.20)
Yes	166 (7.76)	71 (7.80)
**Surgery of primary site**		
No	1225 (57.30)	519 (57.03)
Yes	913 (42.70)	391 (42.97)

### Univariate and multivariate analysis of prognostic factors in the training set

Table 2 shows the univariate analysis of demographic and clinicopathological factors investigated and final multivariate results for OS of LCNEC patients in the training cohort. In the univariate analysis, significant variables for OS included age at diagnosis (*P* < 0.001), gender (*P* < 0.001), primary site (*P* < 0.001), laterality (*P* = 0.017), tumor stage (*P* < 0.001), T Stage (*P* < 0.001), N stage (*P* < 0.001), M stage (*P* < 0.001), tumor size (*P* < 0.001), separate tumor nodules in the ipsilateral lung (*P* < 0.001), surgery to other regional and distant sites (*P* < 0.001), and surgery of primary site (*P* < 0.001).

**Table 2:** Univariate and multivariate analyses of variables for overall survival (OS) in the training set.

	Univariate analysis			Multivariate analysis		
	*P*	HR	95% CI	*P*	HR	95% CI
**Age at diagnosis**						
< 50	Reference			Reference		
50–59	0.110	0.821	0.645–1.046	0.457	1.098	0.859–1.402
60–69	0.739	0.962	0.767–1.208	0.197	1.316	1.045–1.659
70–79	0.349	1.115	0.888–1.401	**5.27E-06**	**1.720**	**1.362–2.173**
>80	**7.01E-06**	**1.792**	**1.389–2.311**	**2.62E-11**	**2.428**	**1.871–3.151**
Gender						
Female	Reference			Reference		
Male	**9.09E-05**	**1.221**	**1.105–1.350**	**1.50E-05**	**1.255**	**1.132–1.390**
**Race**						
Black	Reference					
White	0.190	1.112	0.949–1.303			
Other	0.884	0.978	0.727–1.317			
**Primary site**						
Main bronchus	Reference			Reference		
Upper lobe	6.13E-13	0.417	0.328–0.529	0.1.64	0.814	0.635–1.045
Middle lobe	6.92E-05	0.517	0.374–0.716	0.691	0.934	0.667–1.308
Lower lobe	1.18E-10	0.442	0.345–0.567	0.120	0.814	0.628–1.055
Overlapping lesion of lung	0.083	0.634	0.379–1.062	0.990	1.003	0.595–1.691
Lung, NOS	0.825	0.9663	0.713–1.309	0.931	1.015	0.734–1.403
**Histologic grade**						
I (Well differentiated)	Reference					
II (Moderately differentiated)	0.253	0.617	0.270–1.411			
III (Poorly differentiated)	0.902	0.957	0.476–1.924			
IV (Undifferentiated; anaplastic)	0.874	1.059	0.522–2.150			
Unknown	0.162	1.643	0.819–3.297			
**Laterality**						
Left	Reference			Reference		
Right	0.217	1.066	0.963–1.180	0.560	1.032	0.928–1.148
Other	**0.017**	1.744	**1.110–2.835**	0.109	0.642	0.373–1.105
**Tumor stage**						
I	Reference			Reference		
II	**8.50E-06**	**1.617**	**1.309–1.998**	**0.015**	**1.371**	**1.062–1.768**
III	**<2E-16**	**2.650**	**2.263–3.104**	**0.008**	**1.367**	**1.083–1.726**
IV	**<2E-16**	**5.832**	**5.078–6.698**	**9.27E-08**	**2.720**	**1.883–3.926**
**T stage**						
T1	Reference			Reference		
T2	**4.70E-05**	**1.330**	**1.160–1.527**	0.518	0.942	0.787–1.129
T3	**<2E-16**	**2.127**	**1.798–2.516**	0.983	1.003	0.789–1.275
T4	**<2E-16**	**3.179**	**2.753–3.670**	0.405	1.093	0.886–1.348
Tx	**1.23E-15**	**3.534**	**2.594–4.815**	0.543	0.895	0.627–1.279
**N stage**						
N0	Reference			Reference		
N1	**3.71E-08**	**1.647**	**1.379–1.967**	0.068	1.213	0.986–1.492
N2	**<2E-16**	**2.608**	**2.319–2.934**	**0.002**	**1.287**	**1.101–1.505**
N3	**<2E-16**	**3.816**	**3.242–4.493**	**1.38E-05**	**1.532**	**1.264–1.857**
Nx	**9.92E-16**	**4.149**	**2.931–5.872**	**0.013**	**1.597**	**1.105–2.307**
**M stage**						
M0	Reference			Reference		
M1a	**<2E-16**	**3.887**	**3.107–4.864**	0.979	1.005	0.692–1.461
M1b	**<2E-16**	**3.832**	**3.433–4.278**	0.447	1.1287	0.826–1.542
M1, NOS	**2.61E-15**	**3.340**	**2.477–4.503**	NA	NA	NA
**Tumor size**						
<20 mm	Reference			Reference		
20–29 mm	**0.002**	**1.303**	**1.100–1.543**	0.111	1.150	0.968–1.366
30–39 mm	**4.48E-08**	**1.623**	**1.364–1.930**	0.511	1.070	0.874–1.310
40-49mm	**1.86E-05**	**1.514**	**1.252–1.831**	0.434	1.093	0.874–1.369
> = 50 mm	**<2E-16**	**2.331**	**2.008–2.707**	**0.017**	**1.264**	**1.042–1.532**
**Separate Tumor Nodules—Ipsilateral Lung**						
No separate tumor nodules	Reference			Reference		
Separate tumor nodules in different lobe of ipsilateral lung	**4.59E-13**	**2.475**	**1.936–3.164**	0.475	1.104	0.842–1.449
Separate tumor nodules in same lobe of ipsilateral lung	**1.92E-06**	**1.692**	**1.363–2.101**	0.320	1.140	0.881–1.475
Separate tumor nodules, NOS	**2.50E-12**	**2.556**	**1.965–3.323**	0.745	1.048	0.790–1.390
Unknown	5.49E-01	1.034	0.926–1.155	0.933	0.995	0.885–1.390
**Surgery to Other Regional/Distant Sites**						
No	Reference			Reference		
Yes	**1.09E-04**	**1.418**	**1.188–1.692**	0.072	0.841	0.696–1.016
**Surgery of primary site**						
No	Reference			Reference		
Yes	**<2E-16**	**0.250**	**0.224–0.280**	**2.69E-14**	**0.540**	**0.461–0.633**

HR = hazard ratio; CI = confidence interval; NOS = not otherwise specified.

These prognostic factors of statistical significance in the univariate models were included in the multivariate analysis. The factors that retained significance were age at diagnosis (*P* < 0.001), gender (*P* < 0.001), tumor stage (*P* < 0.05), N stage (*P* < 0.05), tumor size (*P* < 0.05), and surgery of primary site (*P* < 0.001).

### Nomogram development and validation

**Fig 1:**
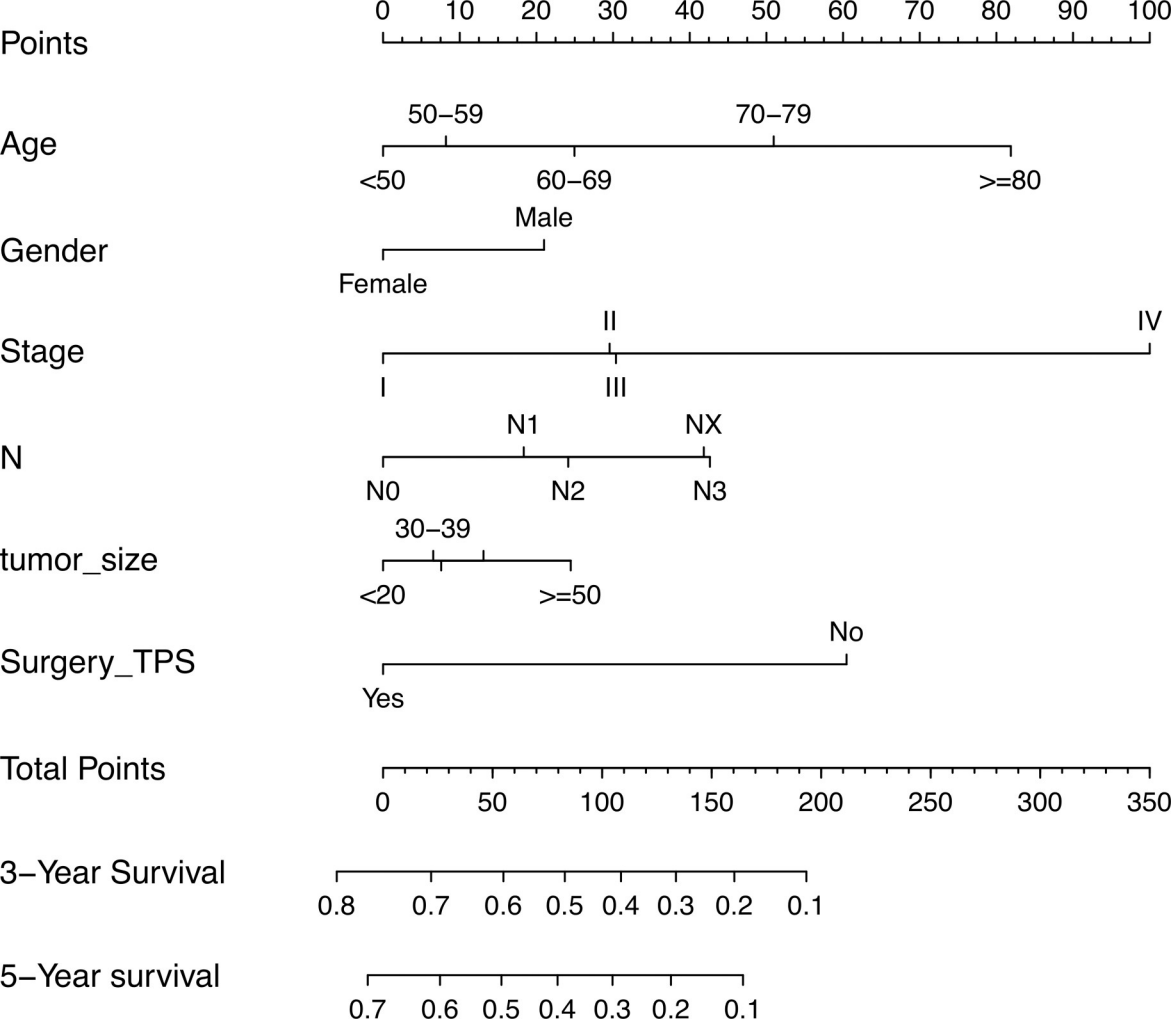
Nomogram predicting 3- and 5-year OS of patients with LCNEC. The nomogram summed the points identified on the scale for each variable. The total points projected on the button scale indicate the probabilities of 3- and 5-year OS.

**Fig 2:**
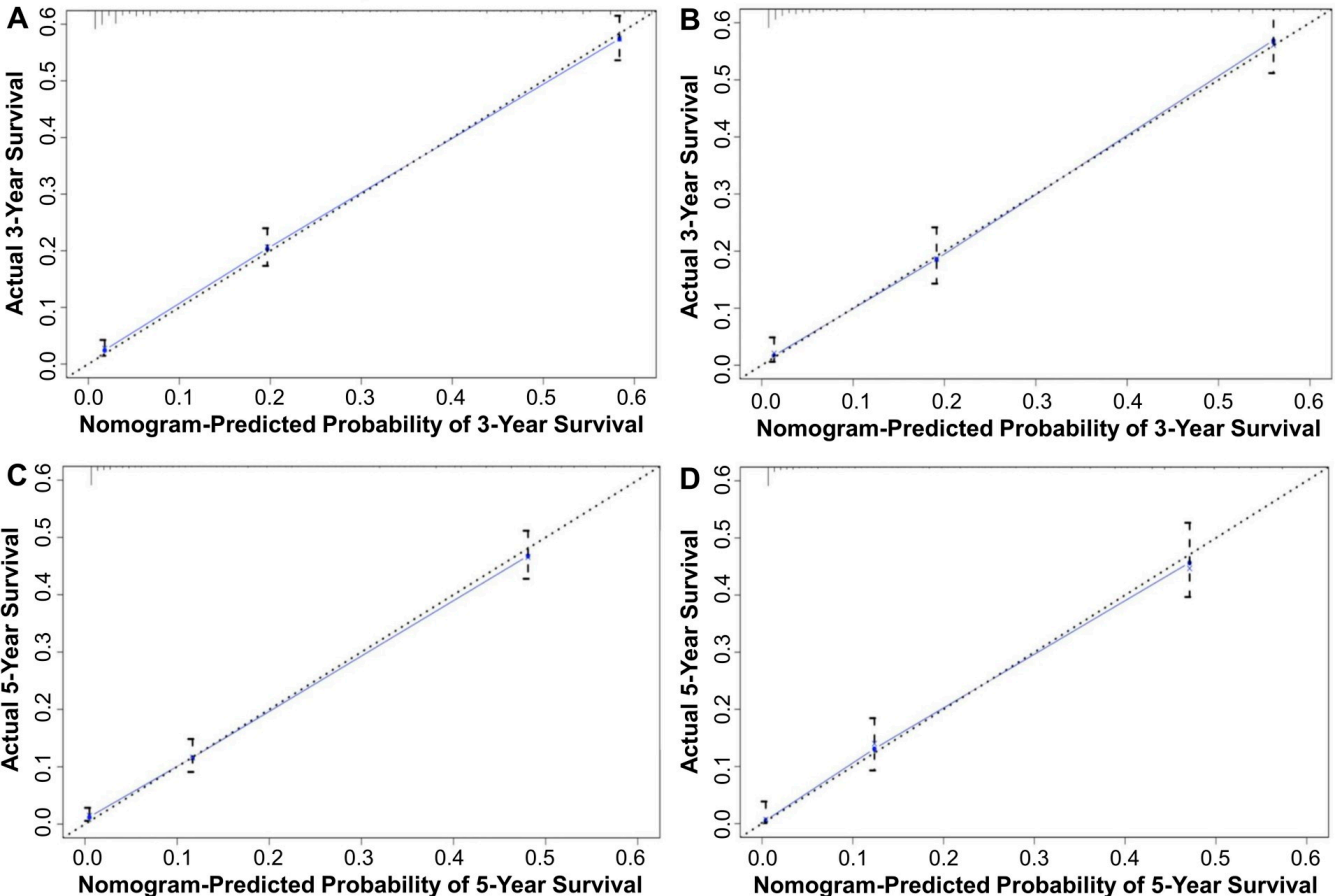
Calibration curves of the nomogram for predicting 3-year OS in the (A) training set and (B) validation set, and predicting 5-year OS in the (C) training set and (D) validation set. Nomogram-predicted OS is plotted on the *x*-axis and the actual OS is plotted on the *y*-axis. The diagonal dotted line indicates the ideal nomogram, in which the actual and predicted probabilities are identical. The solid line indicates the actual nomogram, of which a closer fit to the dotted line indicates a better calibration.

Fig 1 shows the nomogram for predicting the survival of LCNEC using the prognostic factors that were found significant in the multivariate analysis of the training set. The top three factors contributing to LCNEC prognosis were tumor stage, age at diagnosis, and surgery of primary site. Score assignment to each variable included in the nomogram is provided in Table 3. A total score was computed by summing individual score according to demographic and clinical features of individual patients and the patient’s probability of 3- and 5-year OS was obtained from the nomogram (Fig 1). The C-index values for OS prediction were 0.75 (95% CI, 0.74–0.76) and 0.76 (95% CI, 0.74–0.77) in the training and validation sets, respectively. The calibration plots for OS probability at 3 and 5 years showed that the concordance between the predicted and observed survival was optimal in both cohorts (Fig 2).

**Table 3:** Score assignment for each variable included in the nomogram.

Variables	Points
**Age at diagnosis**	
< 50	0
50–59	8
60–69	25
70–79	51
>80	82
**Gender**	
Female	0
Male	21
**Tumor stage**	
I	0
II	30
III	30
IV	100
**N stage**	
N0	0
N1	18
N2	24
N3	43
Nx	42
**Tumor size**	
<20 mm	0
20–29 mm	13
30–39 mm	7
40-49mm	8
> = 50 mm	24
**Surgery of primary site**	
No	60
Yes	0

### Kaplan-Meier analyses

**Fig 3:**
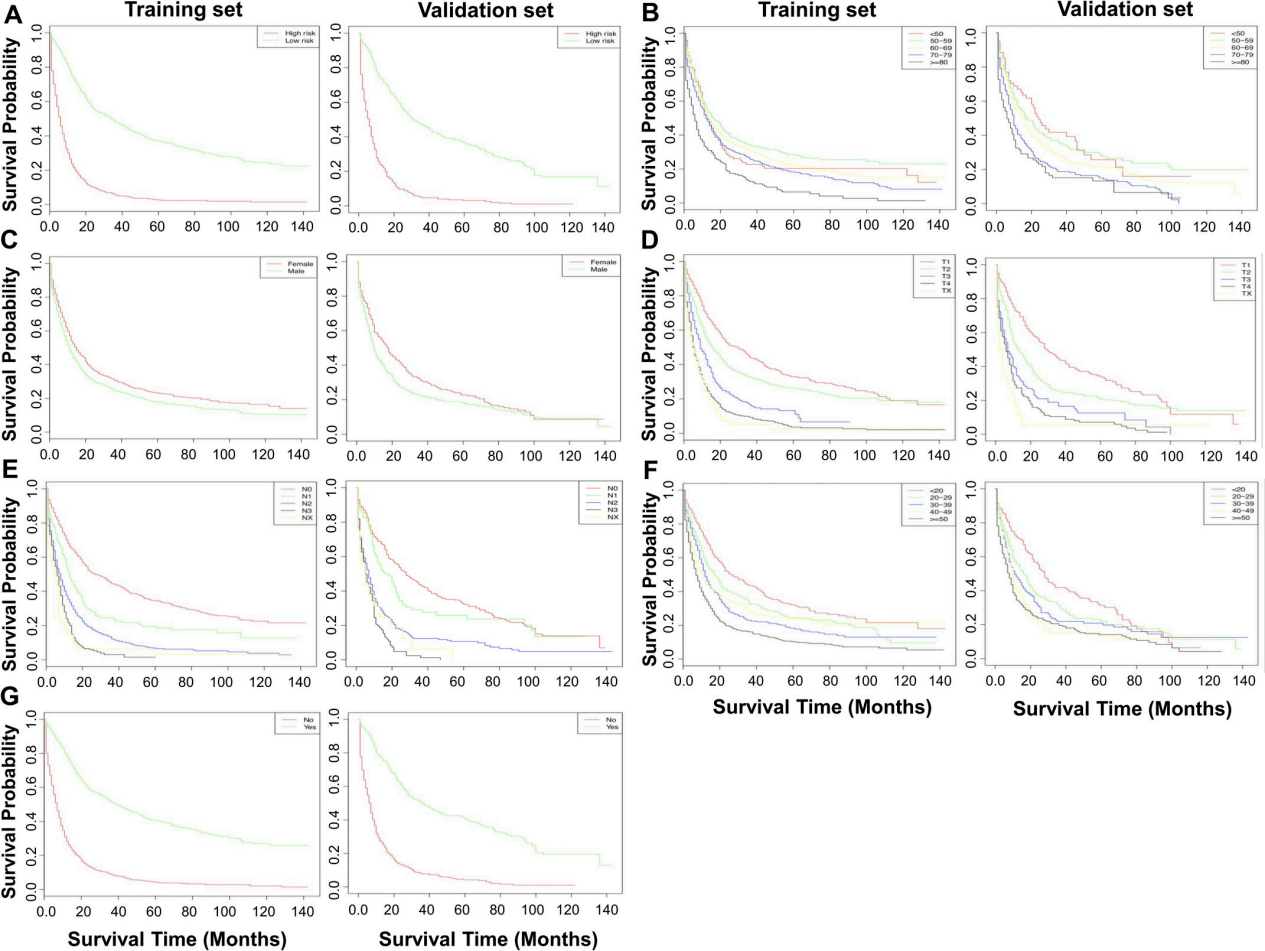
Kaplan-Meier curves of OS in the training and validation sets by (A) risk score, (B) age at diagnosis, (C) gender, (D) tumor stage, (E) N stage, (F) tumor size, and (G) surgery of primary site.

In the training cohort with a median (range) follow-up of 65.5 (1–143) months, Kaplan-Meier analysis revealed a median OS of 13 (95% CI, 12–14) months. In the validation cohort with a median (range) follow-up of 56 (1–143) months, the median OS of LCNEC was 12 (95% CI, 10–15) months. We predicted a risk score based on the independent prognostic factors that were determined significant in the multivariate analysis. Patients with LCNEC were subsequently apportioned to high- and low-risk groups, according to the median risk score of 1.275. Fig 3A shows the Kaplan-Meier curves by the risk score group and the results clearly indicated that this risk score was capable of distinguishing OS of patients with LCNEC (*P* < 2*e*^–16^). In addition, the Kaplan-Meier curves showed worse OS for patients with LCNEC at advanced age (>80 years), male gender, large tumor size (20–29, 40–49, and ≥50 mm), lymph node metastasis, advanced stage (stage II-IV vs I), and not receiving surgery of the primary site (Fig 3B–3G).

## Discussion

We used SEER data to develop and validate a novel nomogram model for predicting OS for patients with LCNEC. To our best knowledge, this prognostic nomogram is the first developed for pulmonary LCNEC. The nomogram incorporated factors that were identified in a multivariate Cox analysis as independent prognostic factors for LCNEC, specifically, age at diagnosis, gender, tumor stage, N stage, tumor size, and surgery of primary site. The nomogram model exhibited high discriminative accuracy in the training cohort (C-index = 0.75) which was confirmed in the validation cohort (C-index = 0.76). In addition, the calibration plots confirmed good concordance for the prediction of OS at 3 and 5 years in both cohorts, suggesting excellent performance of this nomogram for estimating LCNEC prognosis.

We took a population-based approach using the SEER program to develop the nomogram. SEER collects incident cancer cases from cancer registries representing approximately 28% of the United States population [17]. Because of the rarity of LCNEC, this nomogram study would be impossible if based on cases of a single institution or multiple institutions. Furthermore, SEER is the only population-based program in the United States that provides follow-up information and comprehensively documents clinical data from medical records, including stage of cancer at diagnosis, grade, and therapy [18]. Given the robustness and completeness of the SEER database, the nomogram developed in this study can be expected to represent patients in the United States, with potential universal application for all patients with LCNEC.

Currently, the criterion for assessing prognosis of neuroendocrine tumors is the TNM staging system, recommended by the IASLC [1, 7]. However, the effectiveness of this system is unclear; results from small studies have showed conflicting results regarding the predictors for prognosis of LCNEC. LCNEC histology is in general related to worse OS, but even for stage I LCNEC, patients receiving adjuvant chemotherapy achieved better OS than those receiving surgery alone [19]. In another single-institution retrospective study, the survival benefit of adjuvant therapy was apparent in patients with LCNEC stages II or higher, but negligible in patients with stage I [20]. However, due to limited sample size and the lack of generalizability of these studies, no definitive and sound conclusion has been drawn.

With access to the SEER data, our study comprises the largest population of LCNEC ever studied. The multivariate analysis indicated that the tumor features significantly associated with patient survival were tumor stage and N stage. The classic T and M stages failed to attain independent prognostic significance. Tumor stage spread through the full range of the point axis and contributed the most points in the nomogram, suggesting a more significant influence of tumor stage on LCNEC prognosis. Our study clearly suggests that the traditional TNM staging system may be insufficient for predicting prognosis of LCNEC. Surprisingly, we failed to find a significant effect on survival for histologic grade. This may be related to the high proportion of patients with unknown tumor grade.

Age at diagnosis was second to tumor stage to extend across most range of the point axis in the nomogram. Both the univariate and multivariate analyses showed that being 80 years and older was associated with a poorer OS compared with younger ages; the OS of patients aged 70 to 79 years was similar to that of younger patients. This is somewhat inconsistent with previous studies. In a recent retrospective study of LCNEC, the factors found to significantly contribute to poor survival were old age (>70 years), male gender, white race, and larger tumors (>30 mm) [9]. Older age (median, 65 years) has also been reported in other studies as a predictor of poor prognosis [4, 21]. Our study also showed that tumor size larger than 50 mm was an independent prognostic factor associated with poor survival in patients with LCNEC. We also showed, for the first time, that male patients had poor OS, and LCNEC is often associated with male gender. Although exposure to heavy smoking is a possible prognostic factor for LCNEC, we were unable to assess its possible contribution to LCNEC survival because SEER does not collect smoking data on individual patients. Future large-scale studies are needed to address this topic.

LCNEC is rare, and the available evidence is insufficient to tailor an optimal treatment plan specified for patients with LCNEC. Primary surgery remains the standard treatment for patients with stage I-II disease, but not all patients with stage I and II LCNEC benefit from surgery. In fact, the 5-year OS of patients with stage I disease who receive only surgery is low [22]. In the present multivariate analysis, LCNEC treated with surgical resection of the primary site was protective, and associated with better OS independently from other variables.

Metastatic patterns and their prognostic value in patients with LCNEC have been investigated in several previous studies, and the most common distant metastatic sites are the bone, liver, brain, and lung [22–24]. Metastasis to distant sites implies poor prognosis, and therefore special attention should be given to clinical management of distant metastases. Our analysis showed that OS in patients with surgery to distant sites was worse compared with those with no surgery to a distant site; the significance disappeared in the multivariate analyses. However, we could not exclude the possibility of lack of significance due to small sample size, especially in the surgery group.

The nomogram provides a personalized estimate of survival. Clinicians can use the total points provided by the nomogram to distinguish high-risk individuals from the general patient population, and pay closer attention during follow-up visits. High-risk patients could be selected to receive more aggressive treatment, or adjustments in treatment in response to changes in tumor features, or a recommendation for clinical trials of systemic therapy. In addition, palliative care service that includes symptom control and psychological support would benefit these patients at high risk for poor prognosis. This nomogram is a useful tool to identify patient subgroups with homogeneous OS within the LCNEC group and potentially assist personalized therapy. Additionally, this nomogram may be used as a prognostic tool to better counsel patients.

This study has several limitations. First, the nomogram was constructed based on the clinicopathological characteristics collected from the SEER database, and thus may not be a comprehensive prediction model for LCNEC prognosis. Mutational landscape differences have been observed between and within histological subtypes of lung cancer and have challenged the traditional histological classification [25]. Incorporating mutational landscape into the nomogram to improve the accuracy of predicting prognosis of LCNEC is promising, but the evidence is preliminary. Some studies advocate for further classification of LCNEC into mutually exclusive subtypes based on mutational signatures [26, 27]. This is supported by recent studies indicating the potential role of mutational signatures in predicting therapeutic response to chemotherapy and prognosis for LCNEC [28, 29]. In this study, we could not provide more insight into the mutational landscape related to LCNEC prognosis, because currently SEER does not capture information pertaining to tumor genetic signatures. The diagnosis of LCNEC requires confirmation of neuroendocrine differentiation, which is recognized by positive immunohistochemical (IHC) stains for CD56, chromogranin A, and synaptophysin. These neuroendocrine biomarkers have shown potential prognostic value in patients with lung cancer [30]. Similarly, since information regarding IHC profiles of these markers is not provided in the SEER database, evaluation and incorporation of these markers into the nomogram was not possible. Additionally, the lack of some clinicopathological data in the SEER database, such as smoking status, comorbidities, family history of cancer, and performance score, hampered our ability to assess these features in relation to LCNEC prognosis. Second, SEER does not contain specific details on treatment regimens, which limited our ability to evaluate further the effect of treatment on LCNEC survival. Third, to validate nomograms, both internal and external validation sets are recommended, but only internal validation was applied in this study. This may weaken the generalizability of the results [11, 31]. Therefore, before this nomogram can be implemented in a clinical setting, additional validation in an independent patient population is needed. Finally, the retrospective nature of SEER data may create a selection bias. Regardless of these inherent limitations, it is generally accepted that data in the SEER database is high quality, and SEER is the most comprehensive database possible for the objective of the current study.

## Conclusion

In this study a novel nomogram was developed and validated based on only six common demographic and clinicopathological variables. The nomogram can be used to individualize prediction of OS for LCNEC. This should facilitate clinical decision making at individualized level. More studies are needed to verify the generalizability of this nomogram, and for improvements that might incorporate the factors that could not be investigated in the present study.
